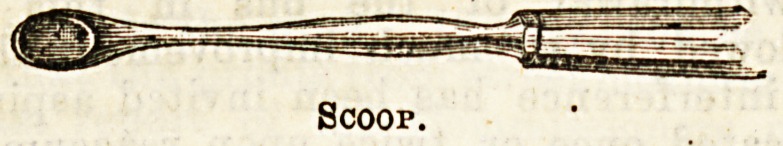# The Treatment of Conjunctivitis

**Published:** 1893-02-18

**Authors:** 


### ROYAL OPHTHALMIC HOSPITAL, MOORFIELDS.

### The Treatment of Conjunctivitis.

For purposes of treatment conjunctivitis may be conveniently considered under two main divisions, namely, acute cases and those which are chronic.

I. First, then, *Acute Cases.* These may be simple, and due to such causes as the introduction of a foreign body, to cold winds, smoke, &c. Their treatment at Moorfields is simply to remove, in the first instance, the cause of the trouble, and then to advise bathing the eye three or four times a day with boracic acid lotion (gr. x. ad. ʒj.), while a little Vaseline or Unguentum Cetacei is put along the edges of the lids at night to prevent them sticking, and so keeping in the discharge. The same treatment is used in cases which arise in the course of eczema of the face, erysipelas, or measles.

II. A more important class of acute cases is *Muco-Purulent Conjunctivitis,* and one important thing to be remembered, so that the patient may be warned, is the fact that the trouble is contagious, and on this account the patients, who are frequently children, are prevented from using the towels used by others, which are a fertile source for spread of the disease. A lotion is then ordered of Perchloride of Mercury 1-6,000, with which the eye is washed out several times a day, or, what is preferred by some, nitrate of silver, gr. ij. to water ʒi., two or three drops to be put in the eye three or four times a day, just after it has been washed out with boracic lotion. Some form of ointment is used along the edges of the lids at night, as in simple cases. Under either of these treatments the trouble usually speedily subsides.

III. But much the most dangerous, and so most important, of the acute inflammations, are the cases of *Purulent Ophthalmia,* for it is by this form of inflammation, when introduced, that large numbers of eyes have been lost in early infancy, the circulation of the cornea becoming strangulated by the acuteness of the inflammatory process, sloughing and general suppuration of the globe then following, ending in total loss of the eye. The greatest care has to be taken in these cases to prevent the spread of the disease to others. The next point is to get the discharge frequently and thoroughly removed, and for this purpose boracic acid lotion is ordered, and the eyes to be washed out with it at first every hour or two hours ; then every morning the infant has to be brought to the hospital, and is seen by the house surgeon, who thoroughly cleanses the eye with lotio hydrargyri perchloridi 1-6,000 or boracic lotion, and then paints the conjuntiva with nitrate of silver of a strength of from ten to twenty grains to the ounce, or else touches it over with the mitigated stick (nitrate of silver one part, nitrate of potash two parts). Silver lotion gr. ij. ad. ʒi. is also sometimes given to the patients to be dropped in three or four times a day, after careful cleansing. This treatment carried out with care is usually very successful, especially if the case comes under observation fairly soon after the onset of the disease. Purulent or gonorrhœal ophthalmia in adults is a still more formidable affair, and very destructive to the eye which it attacks. The cases are not very common fortunately, and when they do occur they almost always have to be taken into the hospital, though the fear of infection is so great that the surgeon and house surgeon are loath to have such a case in, if it could be avoided. If only one eye be attacked, the first point looked to is the protection of the sound eye, and this is done by covering it with a watch glass, made to adhere to the skin of the forehead and nose by means of india-rubber plaster. Especial care is taken at the nose, where naturally the danger is greatest, and an opening must be left at the outer canthus for ventilation, and even then the watch glass gets very hazy by evaporation from the conjunctival sac. This protection, if properly applied, is usually efficient. The next step is to get the discharge frequently and completely removed, which end is attained by washing out with an irrigator every hour, or at the longest, every two hours; especial care must be taken to clear out the upper and lower fornices of the conjunctiva, as here the pus tends to coagulate and accumulate in small masses if special attention be not paid to it. The lotion used is either perchloride of mercury 1 to 6,000, or boracic lotion. After the washing, the conjunctiva is brushed over with nitrate of silver, ten or twenty grains to the ounce. The eye must on no account be tied up, and so the discharge kept in.

Let us now speak of the *Chronic Cases.* In many of these cases no very special cause is noticeable, except perhaps some enlargement of the follicles in the palpebral conjunctiva, and these cases are treated by various forms of astringent lotion. A very favourite one is zinc sulphate gr. ij. ad. ʒi , this may be used as a lotion or as drops, to be put in after the use of boracic lotion ; in either case a little ointment is put along the edges of the lids at night. Other lotions are guttæ cupri sulphatis, gr. j., ad. ʒj.; or lotio aluminis (alum gr. iv. to x., water ʒj.). If the trouble persists in spite of this treatment, then it is often brought to an end by painting with a weak solution of silver nitrate, or applying lapis divinus (copper sulphate 1, alum 1, nitrate of potash 1, formed into sticks) to the lids. In gouty people, on everting the lids, a small lithic acid concretion may sometimes be found, which is responsible for the mischief. This is then removed with the point of a flattened needle, after instilling a little 2 per cent. cocaine to prevent the pain. Should an enlarged meibomian cyst be the cause, this may be easily removed as follows : The lid is everted so as to expose the cyst from the inside, a small grain of solid cocaine is then placed on the swelling, or, what is perhaps more usual, cocaine has been instilled a few minutes previously. The cyst is then fairly slit across with a Van Beere's knife, and the contents and cyst wall scooped out with a small scoop made for the purpose. A pad of wool and a bandage is then put on till the next day.

Scoop.

Very often chronic conjunctivitis is due to some form of error of refraction, and in young adults this is most commonly some kind of astigmatism, and it is only by ordering the right glasses that the trouble can be got rid of. This is the case more especially in patients who have to use their eyes much for near work. In older people ordering the right presbyopic glasses will often go a long way towards putting an end to a slight irritation and redness. Ciliary blepharitis, too, is a productive cause. It is generally treated by freely washing with lotio sodœ bicarbonatis gr. x. ad. ʒj., or lotio acidi borici to get off all the crusts, and then applying unguentum hydrargyri oxidi flavæ, dil. gr. xxiv. ad. ʒj., or dilute nitrate of mercury ointment night and morning. Some advise that at night a piece of cotton wool soaked in lead lotion should be laid over the closed lids and kept on as long as possible. The lead lotion must not be allowed to go into the eye for fear of staining the cornea. A constant look-out is always kept on the lacrymal apparatus to see that the sac is not inflamed (dacryo-cystitis), and so allowing an occasional regurgitation of irritating muco-pus, and by this means keeping up the conjunctival inflammation. The treatment adopted is to dilate the canaliculus, or slit it up, as may be necessary; most commonly the lower one is slit, but some always slit the upper. The sac is then treated with frequent washing out and the nasal duct probed, as may be necessary, so as to allow of a free drain into the nose.

The presence of trachana granules on the lids is another cause of a chronic conjunctivitis, and what is worse, of a superficial keratitis. At Moorfields this is commonly seen among the Jews who come for treatment, especially on Saturdays. The routine plan is to give them boracic lotion, and let them come once a week to have the lids brushed over with nitrate of silver (gr. x.—xx. ad. ℥i.), or touched over with lapis divinus, or mitigated stick. Should ingrowing lashes be present, these must be removed, and in obstinate cases of conjunctivitis all parts of the conjunctival sac must be carefully explored for the cause.

One word in closing, which applies to all cases. In no kind of case is the eye bound up, as this only tends to keep in the secretion, which it is one of the main objects of treatment to get rid of. This, of course, does not apply, however, to cases in which other trouble beside conjunctivitis may be present.

## Figures and Tables

**Figure f1:**